# Dual Role for DsbA in Attacking and Targeted Bacterial Cells during Type VI Secretion System-Mediated Competition

**DOI:** 10.1016/j.celrep.2017.12.075

**Published:** 2018-01-29

**Authors:** Giuseppina Mariano, Laura Monlezun, Sarah J. Coulthurst

**Affiliations:** 1 Division of Molecular Microbiology, School of Life Sciences, University of Dundee, Dundee, DD1 5EH, UK

## Abstract

Incorporation of disulfide bonds into proteins can be critical for function or stability. In bacterial cells, the periplasmic enzyme DsbA is responsible for disulfide incorporation into many extra-cytoplasmic proteins. The type VI secretion system (T6SS) is a widely occurring nanomachine that delivers toxic effector proteins directly into rival bacterial cells, playing a key role in inter-bacterial competition. We report that two redundant DsbA proteins are required for virulence and for proper deployment of the T6SS in the opportunistic pathogen *Serratia marcescens*, with several T6SS components being subject to the action of DsbA in secreting cells. Importantly, we demonstrate that DsbA also plays a critical role in recipient target cells, being required for the toxicity of certain incoming effector proteins. Thus we reveal that target cell functions can be hijacked by T6SS effectors for effector activation, adding a further level of complexity to the T6SS-mediated inter-bacterial interactions which define varied microbial communities.

## Highlights

• Type VI secretion systems (T6SSs) are used by bacteria to attack competitors
• Disulfide bond formation by DsbA promotes assembly of an active T6SS in *Serratia*
• DsbA in the target cell is needed for activation of certain incoming T6SS effectors
• This work reveals that T6SS-delivered effectors can hijack target cell functions

## Graphical Abstract

**Figure:**
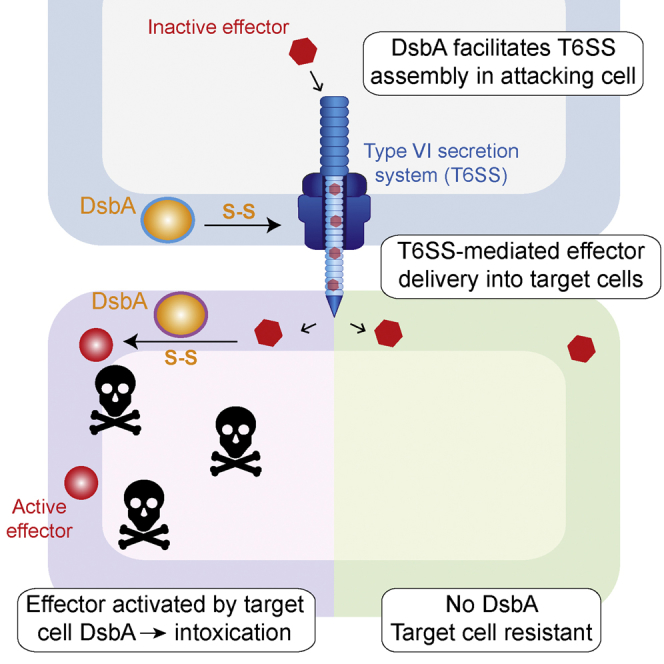

## Teaser

The type VI secretion system is used by many bacteria to deliver toxic effector proteins into rival bacterial cells. Mariano et al. find that DsbA-mediated disulfide bond formation contributes to secretion system function in attacking cells and also to activation of incoming effectors in targeted cells.

## Introduction

Formation of specific disulfide bonds between Cys residues is critical for the function or stability of numerous extra-cytoplasmic and secreted proteins. In Gram-negative bacteria, incorporation of disulfide bonds takes place in the periplasmic compartment and is orchestrated by the Dsb machinery, which is well characterized in *E. coli* and has been reviewed previously (Hatahet et al., 2014, Kadokura and Beckwith, 2010). The primary oxidant is DsbA, a periplasmic thiol-disulfide oxidoreductase that donates a disulfide bond to substrate proteins and is subsequently re-oxidized by the membrane protein DsbB. DsbA displays a thioredoxin fold and a CXXC redox-active catalytic motif. When a protein has more than two Cys residues, DsbA may introduce incorrect disulfide bonds; these are rearranged by the disulfide isomerase DsbC, which is maintained in a reduced state by DsbD. The presence of DsbA is critical for virulence in various bacterial pathogens (Shouldice et al., 2011). It has been shown to be required for folding or stabilization of secreted virulence factors, such as cholera toxin, and for biogenesis of adhesion and motility structures. Additionally, components of certain protein secretion systems also require DsbA for function or stability (Jameson-Lee et al., 2011, Shouldice et al., 2011). Secretion systems are specialized machineries used by bacterial cells to deliver proteins to the extracellular environment or directly into target cells, thus determining bacterial interactions with the host, environment, or competitors (Costa et al., 2015).

The Type VI secretion system (T6SS) is widespread in Gram-negative bacteria and used to deliver toxic effector proteins directly into other cells. T6SSs are macromolecular machineries formed by 14 conserved core components (TssA-M, PAAR), often with additional accessory proteins, that assemble to form a membrane-anchored structure partially resembling an inverted bacteriophage tail. According to the current model (Basler, 2015, Brunet et al., 2015, Cianfanelli et al., 2016b, Durand et al., 2015), a basal complex made up of a membrane complex (TssJLM) and a cytoplasmic baseplate (TssEFGK) serves as an anchoring platform for the co-ordinated assembly of an extended contractile sheath (TssBC) around a long spear-like structure (Hcp/TssD, VgrG/TssI, PAAR) in the cytoplasm. Upon contraction of the TssBC sheath, the puncturing spear, comprising a tube of stacked Hcp hexamers tipped with a sharp VgrG-PAAR spike, is propelled out through the basal complex and can breach a neighboring target cell. Since varied effector proteins can associate with components of the expelled spear by covalent or non-covalent interactions, this “firing” event results in the delivery of toxic effector proteins into target cells (Cianfanelli et al., 2016b, Shneider et al., 2013). The T6SS can act against eukaryotic cells as a classical virulence factor or, more widely, against rival bacterial cells as a key weapon in inter-bacterial competition (Alcoforado Diniz et al., 2015, Russell et al., 2014b). Many T6SS-delivered antibacterial effectors have now been identified. These include eight families of cell wall hydrolase (peptidoglycan amidases and glycoside hydrolases), five families of membrane phospholipase, several classes of nuclease, predicted pore-forming toxins, an NAD(P)^+^ glycohydrolase, and others of unknown function (Alcoforado Diniz et al., 2015, Russell et al., 2014a, Whitney et al., 2015). It is noteworthy that the majority of T6SS antibacterial effectors reported to date, including peptidoglycan hydrolases and phospholipases, act in the periplasm of target cells, although it is currently unclear in which compartment(s) in the target cell effectors are released (Vettiger and Basler, 2016). In order to avoid self-intoxication, either within the secreting cell or between neighboring sibling cells, antibacterial effectors are neutralized by specific immunity proteins encoded adjacent to the toxic effector. Immunity proteins are localized in the compartment of action of the toxin and provide protection by binding to it with high specificity (Alcoforado Diniz et al., 2015, Russell et al., 2014a). Although one example of a target cell protein being required for an effector to access the cytoplasm has been reported (Whitney et al., 2015), the use of specific target cell functions for activation of incoming effectors has not yet been described. DsbA-mediated disulfide bond formation represents a good candidate for such a target cell process. However, whether DsbA is required for T6SS function, either for the secretion machinery itself or for effector activation, has not yet been determined.

To investigate the role of DsbA in T6SS-mediated antibacterial activity, we used the T6SS of *Serratia marcescens*, an opportunistic pathogen frequently isolated as a cause of multidrug resistant human infections or as a virulent insect pathogen (Iguchi et al., 2014). The T6SS of *S. marcescens* strain Db10 represents a good model for studying antibacterial T6SS. Nine antibacterial effectors delivered by this system have been described, including two peptidoglycan hydrolases (Ssp1 and Ssp2), a DNase (Rhs2), and a predicted phospholipase (Alcoforado Diniz and Coulthurst, 2015, Cianfanelli et al., 2016a, English et al., 2012). In this study we aimed to define the role of DsbA during the effective deployment of the T6SS against competitor bacterial cells. We report that DsbA is required for proper functionality of the T6SS in the secreting cell, while the presence of a functional DsbA protein in the recipient target cell is necessary for the toxic action of periplasmic-acting T6SS effectors. This work reveals a dual role for DsbA in both the donor and recipient cell during T6SS-mediated inter-bacterial interactions and highlights the important finding that target cell functions can be hijacked for activation of incoming T6SS effectors.

## Results

### Two Redundant DsbA Homologs Are Required for Pleiotropic Phenotypes, Including Virulence, in *S. marcescens*

**Figure 1:**
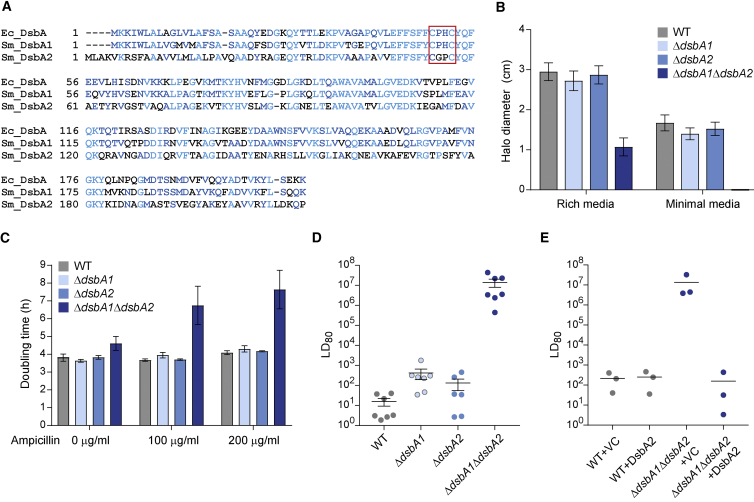
DsbA1 and DsbA2 Are Redundant and Required for Pleiotropic Phenotypes in *S. marcescens* (A) Alignment of the amino acid sequences of DsbA1 (SMDB11_4101) and DsbA2 (SMDB11_0239) from *S. marcescens* Db10 with the single DsbA homolog from *E. coli* K12 (UniProt: P0AEG4). The conserved oxidoreductase catalytic motif (CXXC) is indicated by a red box. (B) Swimming motility of wild-type (WT) or mutant (Δ*dsbA1*, Δ*dsbA2*, and Δ*dsbA1*Δ*dsbA2*) strains of *S. marcescens* Db10 in rich (LB) or minimal media, expressed as mean diameter of the swim halo ± SEM (n = 4). (C) Ampicillin resistance of wild-type and mutant strains, as determined by doubling time during growth in minimal media with differing concentrations of ampicillin. Bars show mean ± SEM (n = 4). (D) Virulence of wild-type *S. marcescens* Db10 and mutant strains in *Galleria mellonella* larvae. Data are expressed as lowest dose of bacteria required to kill 80% of the larvae (LD_80_) after 24 hr (with 20 larvae inoculated per group). Individual data points, overlaid with the mean ± SEM, are plotted for n = 7 independent experiments. (E) Virulence phenotype of the wild-type and the Δ*dsbA1*Δ*dsbA2* mutant carrying the vector control (+VC, pSUPROM) or a plasmid directing the expression of DsbA2 in *trans* (+DsbA2, pSC1507) measured as in (D), except that data are from n = 3 independent experiments. See also Figure S1.

Interrogation of the genome sequence of *S. marcescens* Db10 (Iguchi et al., 2014) revealed the presence of genes encoding two DsbA homologs, SMDB11_4101 (named DsbA1) and SMDB11_0239 (DsbA2). Alignment with the well-characterized single DsbA from *E. coli* revealed that DsbA1 is more closely related to *E. coli* DsbA (71% identity) than DsbA2 (45% identity) (Figure 1A). To investigate the role and potential redundancy of the two homologs, in-frame deletion mutants lacking *dsbA1*, *dsbA2*, or both were generated. The Δ*dsbA1* and Δ*dsbA2* mutants showed no growth difference compared with the wild-type under any condition. The double Δ*dsbA1*Δ*dsbA2* mutant showed no impairment under standard growth conditions (rich media with good aeration) but did exhibit a minor reduction in growth rate under conditions of reduced aeration (96-well plate), particularly in minimal media (Figure S1). Considering phenotypes predicted to be affected by lack of DsbA, swimming motility and ampicillin resistance (dependent on endogenous periplasmic β-lactamases) were assessed in the DsbA mutants. Neither motility nor growth in the presence of ampicillin was affected in the single Δ*dsbA1* and Δ*dsbA2* mutants compared with the wild-type. In contrast, the Δ*dsbA1*Δ*dsbA2* mutant displayed reduced swimming motility in rich media, complete loss of swimming in minimal media, and reduced growth rate in the presence of ampicillin (Figures 1B and 1C). The loss of motility in the Δ*dsbA1*Δ*dsbA2* mutant could be fully complemented by the expression of DsbA2 in *trans*. However, the equivalent overexpression of DsbA1 had a deleterious impact on growth and therefore could not be used for complementation (Figure S1). We speculated that the two *dsbA* homologs might have distinct roles in virulence. Using the *Galleria mellonella* (wax moth) larvae model, the Δ*dsbA1*Δ*dsbA2* mutant was found to be essentially avirulent (LD_80_ [lethal dose to 80% of larvae] value of ∼10^7^ bacterial cells), compared with the wild-type (LD_80_ ∼10 cells). This loss of virulence could be fully complemented by the expression of DsbA2 in *trans* (Figures 1D and 1E). In contrast, although the Δ*dsbA1* mutant had a slightly higher mean LD_80_ value, neither single mutant was significantly impaired compared with the wild-type (p > 0.05, t test). Taken together, these data show that DsbA1 and DsbA2 show a high degree of redundancy, but at least one functional DsbA homolog is required for several important phenotypes, including virulence, in *Serratia marcescens*.

### DsbA Is Required in the Secreting Cell for Full T6SS Function and Acts on Several Components of the Secretion Machinery

**Figure 2:**
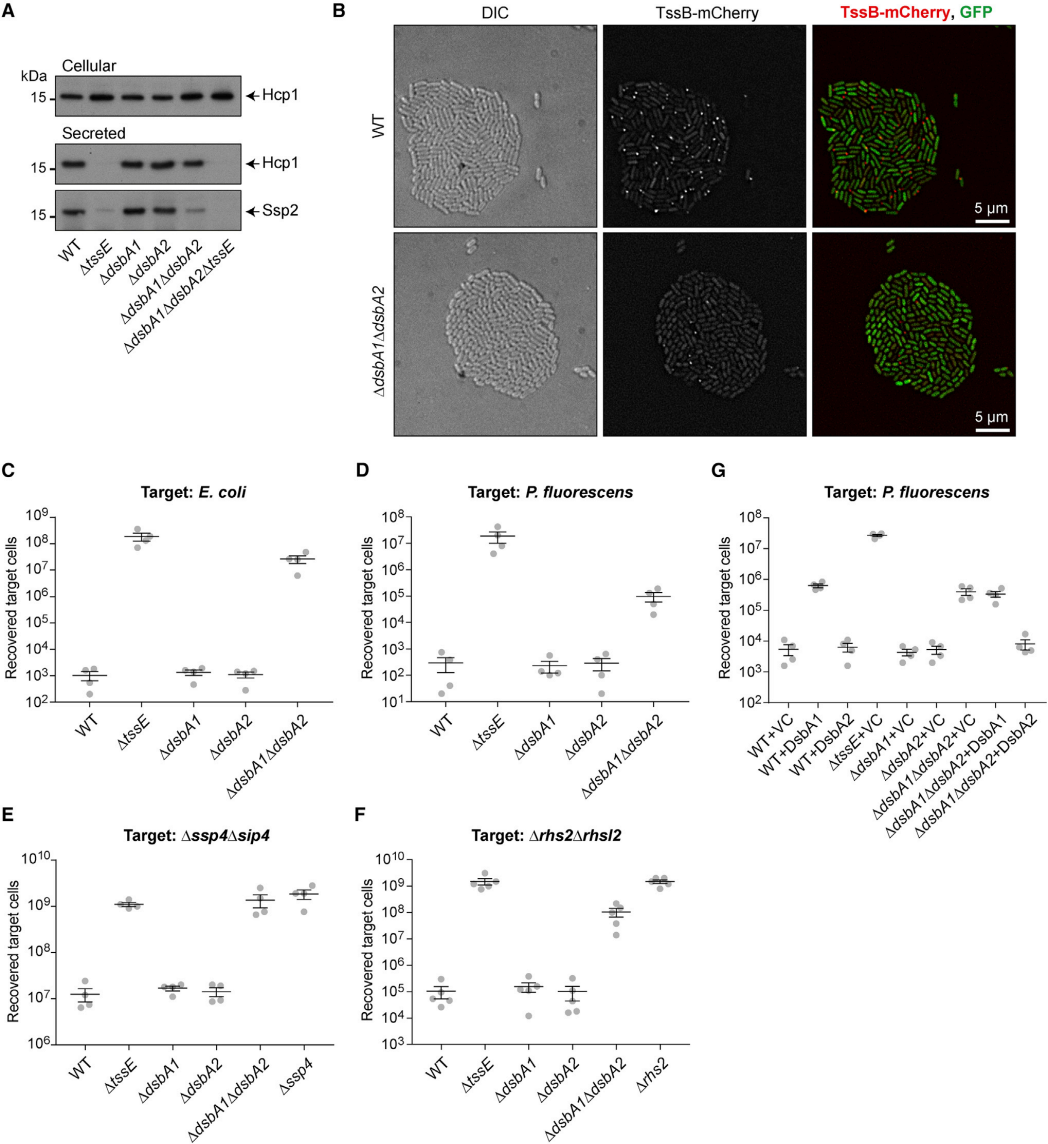
Loss of DsbA1 and DsbA2 Disrupts the Function of the Type VI Secretion Machinery (A) Immunoblot detection of Hcp1 and Ssp2 in the cellular and secreted fractions of wild-type (WT) or mutant (Δ*tssE*, Δ*dsbA1*, Δ*dsbA2*, and Δ*dsbA1*Δ*dsbA2*) strains of *S. marcescens* Db10. The Δ*tssE* mutant represents a T6SS-inactive control. (B) Representative fluorescent microscopy images of the Δ*dsbA1*Δ*dsbA2* mutant and the parental strain (WT) each expressing the TssB-mCherry reporter fusion together with uniform cytoplasmic GFP. From left to right: DIC image, mCherry channel (TssB-mCherry), and a false-colored merge of TssB-mCherry (red) with GFP (green). (C–F) Recovery of target organisms *E. coli* MC4100 (C), *P. fluorescens* (D), Db10 Δ*ssp4*Δ*sip4* (E), or Db10 Δ*rhs2*Δ*rhsI2* (F), following co-culture with wild-type or mutant strains of *S. marcescens* Db10 as attackers. The Δ*ssp4*Δ*sip4* and Δ*rhs2*Δ*rhsI2* target strains are non-immune mutants of *S. marcescens* Db10 sensitive to Ssp4 and Rhs2, respectively. (G) Recovery of *P. fluorescens* following co-culture with wild-type or mutant strains of *S. marcescens* Db10 carrying either the vector control (+VC, pSUPROM) or plasmids directing the expression of DsbA1 (+DsbA1, pSC1506) or DsbA2 (+DsbA2, pSC1507) in *trans*. For (C)–(G), individual data points overlaid with mean ± SEM (n = 4) are shown, except (F) where n = 5.

In order to determine whether DsbA-mediated disulfide bond formation is required within the T6SS machinery, we examined T6SS function in wild-type and *dsbA* mutants of *S. marcescens* Db10. First, the basic ability of the system to “fire” Hcp and effector proteins to the media in liquid culture was determined, revealing that the Δ*dsbA1*Δ*dsbA2* mutant was still able to secrete Hcp and Ssp2, albeit with a reduced efficiency obvious for Ssp2 (Figure 2A). In contrast, single *dsbA* mutants were indistinguishable from the wild-type. The ability of individual cells within a microcolony to assemble a firing-competent T6SS can be determined by observing formation of TssB-mCherry foci (contractile TssBC sheath structures; Gerc et al., 2015). Here, the Δ*dsbA1*Δ*dsbA2* mutant showed a markedly reduced frequency of TssB focus formation compared with the wild-type (Figure 2B). Finally, we examined the impact of *dsbA* deletion on T6SS-mediated antibacterial activity, the most sensitive measure of proper T6SS function. Although the single Δ*dsbA1* and Δ*dsbA2* mutants were again indistinguishable from the wild-type, the Δ*dsbA1*Δ*dsbA2* mutant showed almost no activity against *E. coli* and considerably reduced activity against *P. fluorescens* (Figures 2C and 2D). Similarly, when using non-immune mutants of Db10 susceptible to individual effectors Ssp4 (Figure 2E) or Rhs2 (Figure 2F) as target strains, the Δ*dsbA1*Δ*dsbA2* mutant was greatly impaired. This loss of antibacterial activity could be fully complemented by expression of DsbA2 in *trans* (Figure 2G). However, overexpression of DsbA1 inhibited the activity of the wild-type strain, consistent with its negative impact on growth (Figure S1), suggesting that excess levels of this protein are harmful.

**Figure 3:**
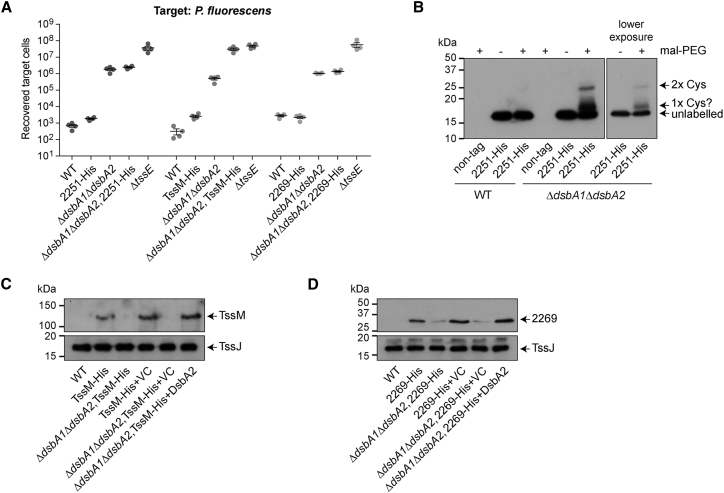
Several T6SS Components Are Subject to the Action of DsbA in *S. marcescens* Db10 (A) Recovery of *P. fluorescens* following co-culture with wild-type *S. marcescens* Db10 (WT), the Δ*tssE* and Δ*dsbA1*Δ*dsbA2* mutants, and strains carrying chromosomally encoded C-terminal His_6_ fusions with SMDB11_2251 (2251-His), TssM (TssM-His), or SMDB11_2269 (2269-His) in the parental or Δ*dsbA1*Δ*dsbA2* mutant background. Individual data points are overlaid with mean ± SEM (n = 4). (B) Mal-PEG labeling of free (reduced) cysteine residues in 2251-His in the parental (WT) or Δ*dsbA1*Δ*dsbA2* mutant background. The 2251-His protein was detected by anti-His_6_ immunoblot; two exposures of 2251-His in Δ*dsbA1*Δ*dsbA2*, with and without labeling, are shown to aid clarity. Non-tag indicates control strains without the 2251-His fusion. (C) Immunoblot detection of TssM-His in membrane fractions from strains carrying the TssM-His fusion, in the parental or Δ*dsbA1*Δ*dsbA2* background, and from these strains carrying either the vector control (+VC, pSUPROM) or a plasmid directing the expression of DsbA2 (+DsbA2, pSC1507) in *trans*. Bottom: immunoblot detection of the Cys-free control protein TssJ and the wild-type strain (WT) is a control with no fusion protein. (D) As in (C), except that 2269-His and TssJ were detected in whole-cell samples of the strains indicated. See also Figure S2.

Given that a functional DsbA homolog is required for proper T6SS activity, we aimed to determine which component(s) of the machinery it acts on. Three T6SS proteins were identified as candidate DsbA substrates, on the basis of the presence of Cys residues within a protein or domain that is known, or predicted, to be periplasmically located: TssM (SMDB11_2255), part of the core membrane complex, and two accessory components of unknown function, SMDB11_2251 and SMDB11_2269. If these proteins are DsbA substrates, their oxidation state should be altered in the absence of DsbA1 and DsbA2. Labeling whole cells with methoxypolyethylene glycol maleimide (malPEG) allows irreversible labeling of free sulfhydryl groups in the periplasm (Koch et al., 2012), causing a shift of 5 kDa for each reduced cysteine labeled. To allow detection, strains encoding each candidate protein fused with a C-terminal His_6_ tag at the normal chromosomal location were generated and their T6SS functionality determined. Introduction of the His_6_ tag in 2251-His and 2269-His had little or no impact on T6SS-dependent antibacterial activity, whereas the TssM-His fusion caused a small reduction of activity in the wild-type background and further impaired the already-compromised T6SS function in the Δ*dsbA1*Δ*dsbA2* background (Figure 3A). SMDB11_2251 is a predicted periplasmic lipoprotein with two Cys residues in addition to the lipidation site. Upon treatment of the strain encoding 2251-His in Δ*dsbA1*Δ*dsbA2* with malPEG, a species with a 10 kDa shift, corresponding to labeling of both reduced Cys residues, could be detected (Figure 3B). No labeled species were detected in a wild-type background, indicating that both Cys residues had been oxidized during DsbA-dependent disulfide bond formation. TssM is an integral membrane protein with a large C-terminal periplasmic domain that contains two Cys residues in *S*. *marcescens*. Labeling of TssM-His with malPEG was unsuccessful because the protein could not be readily detected in the Δ*dsbA1*Δ*dsbA2* mutant. Analysis of total membrane fractions confirmed that levels of TssM were greatly reduced, almost undetectable, in the Δ*dsbA1*Δ*dsbA2* mutant compared with the wild-type and could be restored by expression of DsbA2 in *trans* (Figure 3C). In contrast, levels of TssJ, a periplasmic lipoprotein that is also part of the membrane complex but does not contain any non-lipidated Cys residues, were unaltered. Loss of stability in the absence of DsbA provides indirect evidence that TssM is subject to DsbA-dependent disulfide bond formation (Pugsley et al., 2001). The atomic structure of the C-terminal part of the periplasmic domain of TssM from enteroaggregative *E. coli* has been reported (Durand et al., 2015). Modeling the equivalent region of *S*. *marcescens* TssM suggested that the two Cys residues are located close in the 3D structure, and disulfide formation could occur between them (Figure S2). Similar to TssM, stability of SMDB11_2269, a predicted periplasmic protein with four Cys residues, was greatly reduced in the Δ*dsbA1*Δ*dsbA2* mutant, implying that this protein is also a DsbA substrate (Figure 3D). Therefore the action of DsbA1 or DsbA2 affects three T6SS components, making an important contribution to the proper function of the system.

### The Presence of DsbA in Target Cells Is Essential for the Activation of Incoming Periplasmic-Acting Effectors Ssp2 and Ssp4

**Figure 4:**
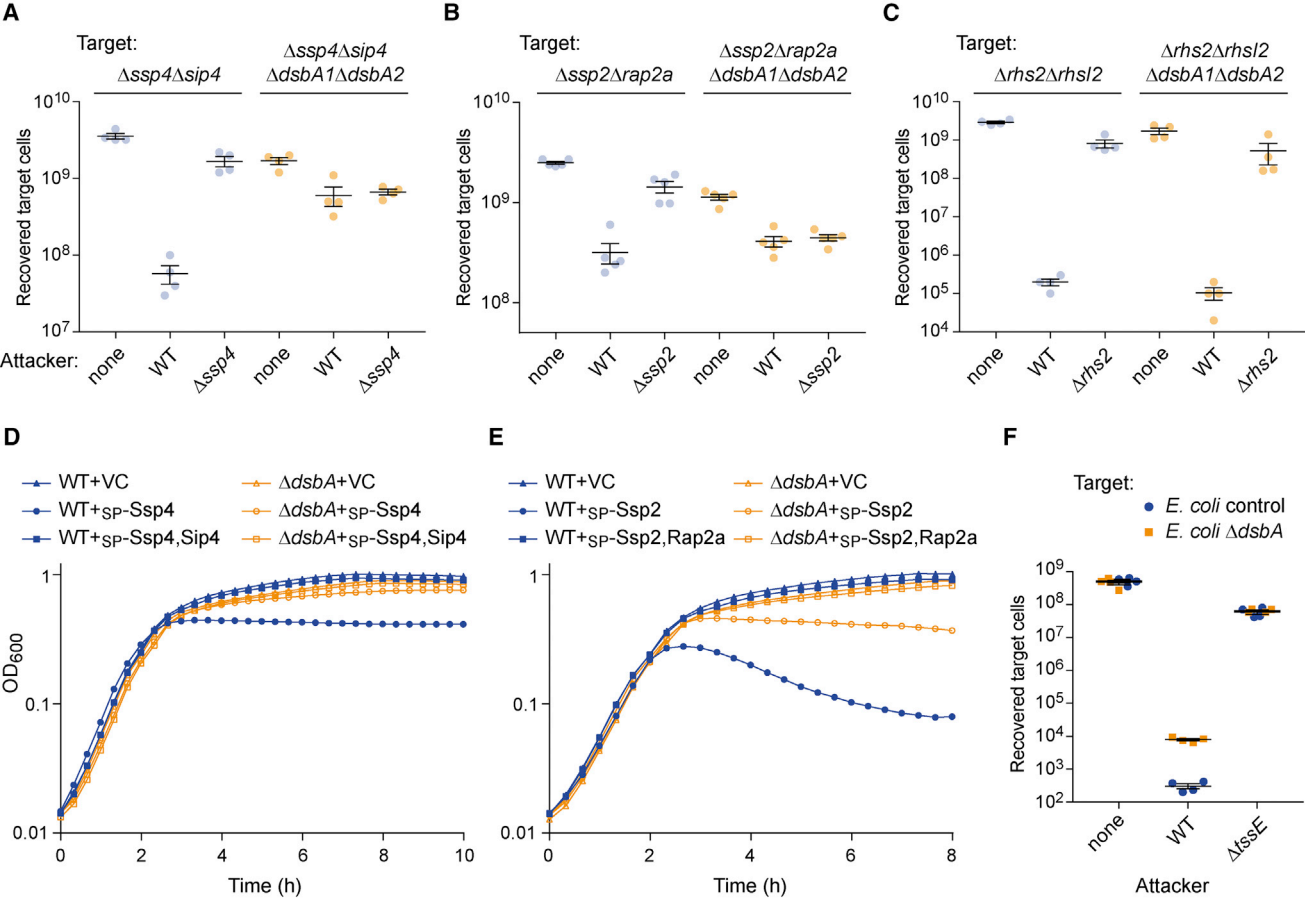
The Presence of DsbA in Target Cells Is Essential for Intoxication by the Periplasmic-Acting Effectors Ssp2 and Ssp4 (A) Recovery of target strains *S. marcescens* Db10 Δ*ssp4*Δ*sip4* and Δ*ssp4*Δ*sip4*Δ*dsbA1*Δ*dsbA2*, following co-culture with wild-type Db10 (WT), the Δ*ssp4* effector mutant, or sterile media alone (none) as attacker. Individual data points are overlaid with the mean ± SEM (n = 4). (B and C) As in (A), except that recovery of the Δ*ssp2*Δ*rap2A* mutant (B) or the Δ*rhs2*Δ*rhsI2* mutant (C), with or without additional Δ*dsbA1*Δ*dsbA2* deletions, following co-culture with the wild-type and the cognate effector mutant was determined. (D) Growth of wild-type (WT) and Δ*dsbA* mutant *E. coli* BW25113 carrying the vector control (+VC, pBAD18-Kn) or plasmids directing the expression of Ssp4 fused with an N-terminal OmpA signal peptide (+_SP_-Ssp4, pSC1234) or _SP_-Ssp4 with Sip4 (+_SP_-Ssp4,Sip4, pSC861). (E) Growth of wild-type and Δ*dsbA* mutant *E. coli* BW25113 when carrying plasmids directing the expression of Ssp2 fused with an OmpA signal peptide (+_SP_-Ssp2, pSC138) or _SP_-Ssp2 with Rap2a (+_SP_Ssp2,Rap2a, pSC144). For (D) and (E), strains were grown in LB with 0.002% arabinose. Points show mean ± SEM (n = 4). (F) Recovery of control (Δ*lacA*::Kn^R^) or Δ*dsbA* (Δ*dsbA*:: Kn^R^) *E. coli* BW25113 following co-culture with wild-type or T6SS-inactive (*ΔtssE*) *S. marcescens* Db10. See also Figure S3.

Because the expelled Hcp-VgrG-PAAR spear, carrying effector proteins, is assembled in the bacterial cytoplasm and propelled directly into the target cell through the T6SS basal complex (Basler, 2015, Durand et al., 2015), effectors should not be exposed in the periplasm of the secreting cell for disulfide bond incorporation by DsbA. On the other hand, we considered whether periplasmic-acting effector proteins might require DsbA-dependent activation in the target cell by the target’s own DsbA proteins. The Ssp2 and Ssp4 toxins are periplasmic-acting effectors delivered by the T6SS of *S. marcescens* Db10 (English et al., 2012, Fritsch et al., 2013) that each contain three Cys residues. They therefore represent good candidates for activation by target cell DsbA. Ssp2 is a Tae4-family amidase toxin that hydrolyses peptidoglycan (Srikannathasan et al., 2013), but the mode of toxicity of Ssp4 is unknown. In order to test whether Ssp4 requires DsbA in the target cell for activity, the susceptibility to Ssp4 of target strains lacking the immunity protein Sip4 in either a wild-type or a Δ*dsbA1*Δ*dsbA2* mutant background was determined. The Δ*ssp4*Δ*sip4* mutant was readily inhibited by delivery of Ssp4, since recovery of the Δ*ssp4*Δ*sip4* target was greatly reduced in the presence of a wild-type compared with a Δ*ssp4* attacker. In contrast, the same target lacking DsbA1 and DsbA2 was entirely resistant to Ssp4, with no difference in recovery between wild-type and Δ*ssp4* attackers (Figure 4A). Similarly, a target strain lacking the immunity protein Rap2a is sensitive to the action of Ssp2 when it possesses DsbA but becomes resistant in a Δ*dsbA1*Δ*dsbA2* background (Figure 4B). In contrast, sensitivity to the cytoplasmic-acting toxin Rhs2 was unaffected in a Δ*dsbA1*Δ*dsbA2* target (Figure 4C). The Δ*dsbA1*Δ*dsbA2* mutant itself does not show any sensitivity to the T6SS of the wild-type, surviving equally well when co-cultured with a wild-type or a Δ*tssE* attacker (Figure S3). To confirm a requirement for DsbA in the toxicity of the effector proteins, rather than only for their entry or release into the target cell, Ssp2 and Ssp4 were artificially expressed and targeted to the periplasm in wild-type or Δ*dsbA* mutant *E. coli* BW25113. In each case, loss of DsbA (or co-expression of the immunity protein) provided relief of toxicity (Figures 4D and 4E). Altogether, these results suggested that a Δ*dsbA* mutant of *E. coli* would be less susceptible to the T6SS of *S. marcescens* than the wild-type. Co-culture assays exposing wild-type or Δ*dsbA* mutant *E. coli* to *S. marcescens* confirmed that this was indeed the case (Figure 4F).

**Figure 5:**
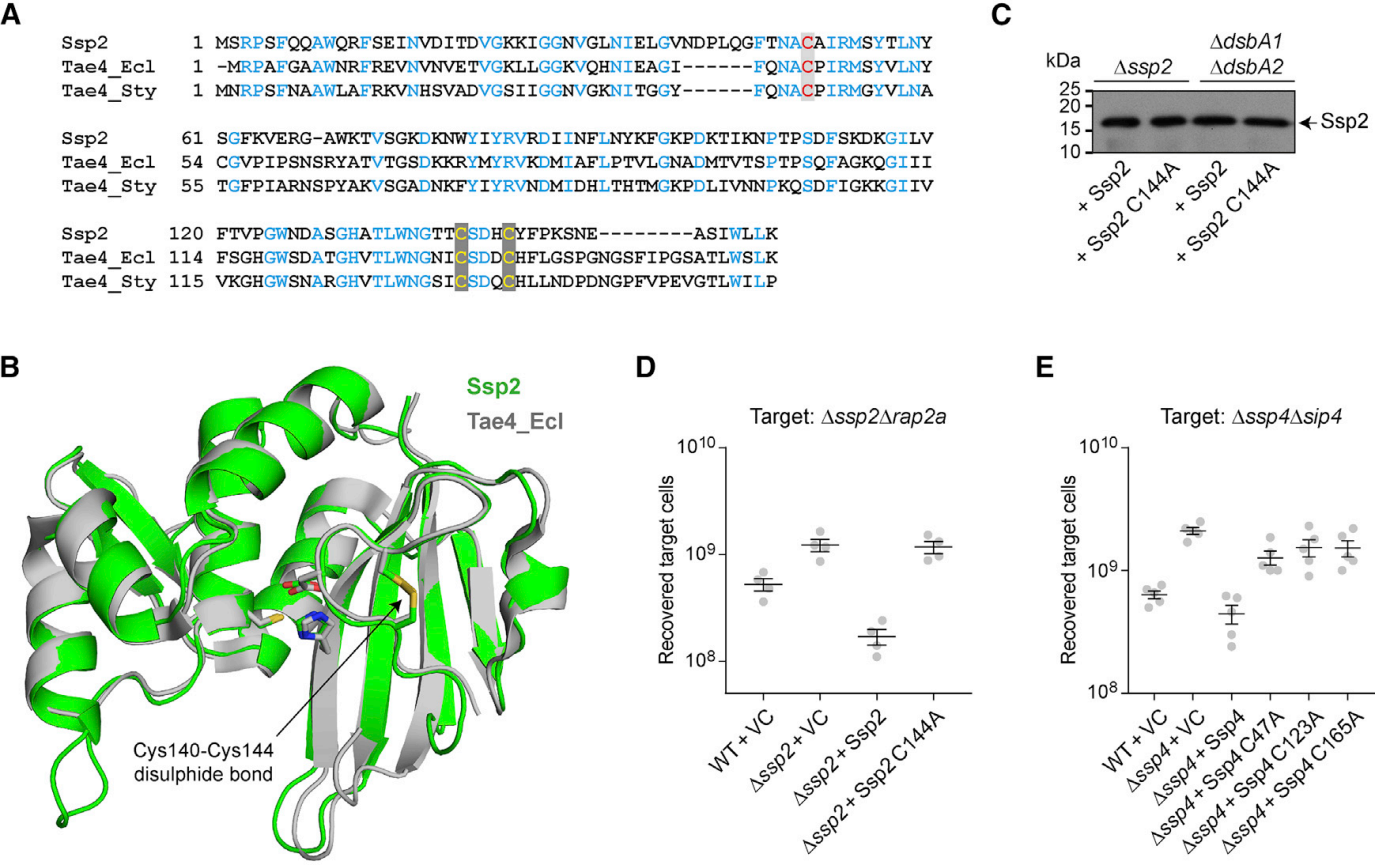
Disulfide Bond Formation in Ssp2 Is Required for Toxicity but Not Stability (A) Sequence alignment of Ssp2 from *S. marcescens* Db10 (SMDB11_2264), Tae4 from *Ent. cloacae* (Tae4_Ecl; UniProt: A0A0H3CIJ2), and Tae4 from *Sal*. Typhimurium (Tae4_Sty; UniProt: Q93IS4). The catalytic Cys residue is shown in red, and the pair of Cys residues indicated to form a disulfide bond are shown in yellow. (B) Structural alignment of Tae4_Ecl (gray) with a model of Ssp2 (green). The Ssp2 model was generated based on the structures of Tae4_Ecl (PDB: 4HFL) and Tae4_Sty (PDB: 4J32) using iTASSER (C score 1.25, TM score 0.89 ± 0.07), with further refinement by molecular dynamics simulation. Residues involved in the disulfide bond (Ssp2 Cys140-Cys144) or catalytic triad (Ssp2 Cys50, His131, Asp142) are shown as sticks. (C) Immunoblot detection of wild-type Ssp2 and Ssp2 C144A in the total cellular fraction of Δ*ssp2* or Δ*dsbA1*Δ*dsbA2* strains of *S. marcescens* Db10. Expression of Ssp2 and Ssp2 C144A was from pSUPROM-based plasmids pSC1588 (+Ssp2) and pSC1589 (+Ssp2 C144A), respectively. (D) Recovery of target strain Δ*ssp2*Δ*rap2a* following co-culture with wild-type (WT) or Δ*ssp2* mutant *S. marcescens* Db10 carrying the vector control (+VC) or plasmids directing the expression of Ssp2 and Ssp2 C144A. Individual data points are overlaid with the mean ± SEM (n = 4). (E) Recovery of target strain Δ*ssp4*Δ*sip4* following co-culture with wild-type or Δ*ssp4* mutant Db10 carrying the vector control (+VC, pBAD18-Kn) or plasmids directing the expression of Ssp4 (+Ssp4, pSC836), Ssp4 C47A (+Ssp4 C47A, pSC1598), Ssp4 C123A (+Ssp4 C123A, pSC1599), or Ssp4 C165A (+Ssp4 C165A, pSC2500). Ssp4 variant expression was induced with 0.002% arabinose. Individual data points are overlaid with the mean ± SEM (n = 5).

The requirement to have a functional DsbA protein in a target cell in order to observe effector toxicity strongly suggests that the effectors contain disulfide bonds in their final, active conformation. Ssp2 contains three Cys residues, one of which, C50, has been shown to be a catalytic residue during peptidoglycan cleavage (Srikannathasan et al., 2013). The other two Cys residues (C140 and C144) are highly conserved in other Tae4-family amidase effectors, including Ssp1 and *Ent. cloacae* Tae4, in which two proteins these Cys residues form disulfide bonds in the atomic structures (Srikannathasan et al., 2013, Zhang et al., 2013). Ssp2 is particularly closely related to *Ent. cloacae* and *Sal.* Typhimurium Tae4 homologs (Figure 5A; Srikannathasan et al., 2013). Modeling Ssp2 based on the structures of these two proteins yields a high-confidence model that displays a structurally conserved disulfide bond between C140 and C144 (Figure 5B). To validate the model, a C144A point mutation was introduced into Ssp2, abolishing any disulfide formation. The C144A mutant was as stable as native Ssp2, and both variants were equally stable between wild-type and Δ*dsbA* mutant cells, consistent with effectors needing to be stable without disulfide bonds prior to secretion. However, the C144A mutant was non-toxic, as shown by its complete inability to complement a Δ*ssp2* mutant for T6SS-mediated antibacterial activity, confirming the requirement for disulfide bond formation in this effector (Figures 5C and 5D). Ssp4 is much less well characterized than Ssp2. Nevertheless, similar to Ssp2, all three of its Cys residues are required for T6SS-mediated antibacterial activity (Figure 5E). This is consistent with the active toxin containing an essential disulfide bond between two of the Cys residues, while the third may have a catalytic function.

## Discussion

This work has revealed a broad and important role for DsbA-dependent disulfide bond formation in *S. marcescens*, being required for phenotypes including motility, virulence, and T6SS-mediated antibacterial activity. *S. marcescens* encodes two DsbA homologs that appear to be entirely redundant, at least for the phenotypes examined. Multiple homologs of DsbA have been reported in other species, in which typically they play different roles in distinct phenotypes (Heras et al., 2010, Jameson-Lee et al., 2011, Sinha et al., 2004). Therefore it remains to be determined whether DsbA1 or DsbA2 of *S. marcescens* have specific functions under particular conditions or during assembly of other cellular structures or whether they simply represent a fail-safe, “belt-and-braces” approach to ensuring correct extra-cytoplasmic protein folding.

DsbA has been reported previously to have a critical role in the proper functioning of components from three classes of protein secretion system, Type II, Type III, and Type IV, including the Type II pseudopilin PulK, the Type III outer membrane secretins YscC and SpiA, and several core components of the *Legionella* Dot/Icm Type IV system (Jackson and Plano, 1999, Jameson-Lee et al., 2011, Kpadeh et al., 2015, Miki et al., 2004, Pugsley et al., 2001). Therefore it was of great interest to discover whether DsbA is also required for the proper function of the T6SS, the other widespread class of large trans-envelope secretion machinery. We show that while DsbA is not essential for the T6SS to fire, absence of both DsbA homologs causes a marked reduction in the number of assembled T6SSs and results in a large reduction in T6SS-dependent antibacterial activity. It should be noted, however, that this loss in T6SS activity is unlikely to represent a significant contribution to the virulence defect of the DsbA mutant, since a T6SS mutant shows no defect in the *Galleria* model (Murdoch et al., 2011).

Given that the T6SS is indeed dependent on DsbA for proper function, we then examined which component(s) are subject to its action in *S. marcescens*. Both the core membrane protein TssM and the predicted accessory protein SMDB11_2269 were found to be highly dependent on the presence of functional DsbA for their stability, which, together with the presence of pairs of Cys residues predicted to be available for DsbA-mediated disulfide bonding in the periplasm, implies they are highly likely to be direct substrates of DsbA. Another predicted accessory protein also encoded within the *S. marcescens* T6SS gene cluster, SMDB11_2251, exhibited DsbA-dependent oxidation, indicating the presence of a DsbA-dependent intramolecular disulfide bond. TssM is an integral inner membrane protein that forms an essential part of the T6SS core membrane complex. The membrane complex recruits the cytoplasmic baseplate, allowing subsequent sheath and spear assembly (Brunet et al., 2015, Durand et al., 2015). Therefore loss of TssM stability should explain some or all of the loss of T6SS function in a DsbA mutant. The residual activity observed suggests either that a small amount of TssM can fold without disulfides or that non-specific oxidation allows a limited amount of spontaneous disulfide formation between the two periplasmic Cys residues. SMDB11_2269 and SMDB11_2251 are also subject to DsbA action, however, their role in the T6SS, and thus the contribution of DsbA through these proteins, remains to be defined. In agreement with our findings, two studies in *Francisella tularensis* identified PdpB, the equivalent of TssM in the highly divergent *Francisella* T6SS, as a potential substrate of the DsbA-like oxidoreductase FipB by using a substrate-trapping approach (Qin et al., 2016, Ren et al., 2014). However, the impact of FipB/DsbA on the function of the T6SS in *F. tularensis* was not examined. The identification of PdpB suggests that TssM can be a conserved target of DsbA. However, TssM is not a universal target, because other homologs, including the well-characterized TssM of EAEC, do not share a pair of periplasmic Cys residues, suggesting that a disulfide bond in TssM may be a system-specific adaptation. Modeling the C-terminal region of TssM from *S. marcescens* based on TssM from EAEC (Durand et al., 2015) showed that formation of a disulfide bond appears to be plausible and would be away from the site of interaction with TssJ (Figure S2). Because Cys 1210 is the penultimate residue of *S. marcescens* TssM, the disulfide bond may serve to stabilize the C terminus of the protein. In contrast, EAEC TssM extends another 28 amino acids beyond the corresponding position, suggesting that the C terminus is configured differently.

Although our data show that DsbA-mediated disulfide bond formation is required for the full activity of the T6SS machinery itself, it is unlikely that DsbA acts on any effectors within the secreting cell. Effectors interact covalently or non-covalently with the T6SS puncturing spear, either within the lumen of the Hcp tube or on the exterior of the VgrG/PAAR spike (Cianfanelli et al., 2016b). This structure is assembled within the cytoplasm and is then fired through the periplasm by an extremely rapid, <2 ms, contraction event (Vettiger et al., 2017). This implies that effectors that ultimately require disulfide bond formation for activity must be stable in the cytoplasm without disulfides, as is observed for Ssp2, even if this is not their final conformation. In contrast, we have revealed here that incorporation of disulfide bonds into incoming effectors by the DsbA protein of the recipient cell can be critical for their toxicity.

There are several possible ways in which target cell functions might be required for effector toxicity. First, target cell proteins may be “hijacked” to allow the effector to reach its compartment of action, similar to how colicins use native outer membrane receptors and periplasmic translocation machinery to be imported into susceptible cells, for example use of BtuB, OmpF, and the Tol-Pal system by ColE2-E9 (Housden and Kleanthous, 2012), or how contact-dependent inhibition systems engage outer membrane receptors such as BamA and then distinct inner membrane proteins to allow nuclease toxin domains to access the cytoplasm (Willett et al., 2015). In the case of the T6SS, the machinery itself allows the target cell to be breached, but it is currently unclear whether effectors are delivered to the periplasm, cytoplasm, or both, by the firing event. Recent evidence indicates that Hcp and VgrG proteins can be delivered to the cytoplasm of the target cell (Vettiger and Basler, 2016), and it has been proposed that certain periplasmic effectors may be able to somehow traffic from the cytoplasm to the periplasm autonomous of the T6SS (Ho et al., 2017). However, there are many reported examples of periplasmic-acting effectors that are not toxic when expressed in the cytoplasm (Flaugnatti et al., 2016, Russell et al., 2012), implying that these effectors cannot independently traffic to the periplasm and must be delivered there by the T6SS. Additionally, the first example of a recipient housekeeping protein being required for a T6SS effector to access its target in the cytoplasm has been reported, namely, the use of EFTu by the NAD(P)^+^ glycohydrolase Tse6 (Whitney et al., 2015). Perhaps most likely is that the T6SS can deliver stochastically to both compartments, but effectors are predominantly released in the periplasm, from which the minority of toxins which act in the cytoplasm use target cell proteins to cross the inner membrane. It is also possible that target cell functions are required for release of effectors from the T6SS machinery, for example, for dissociation from the interior of the Hcp hexamer. Our finding that periplasmic effectors are frequently subject to the action of DsbA is consistent with the hypothesis that disulfide bond formation in the target cell periplasm could be one such means of effector dissociation, leading to a conformational change that can release the effector from Hcp, as proposed previously (Silverman et al., 2013). However, given that DsbA is still required for activity of Ssp2 and Ssp4 when artificially directed to the periplasm in *E. coli*, release from Hcp cannot be its only role.

**Figure 6:**
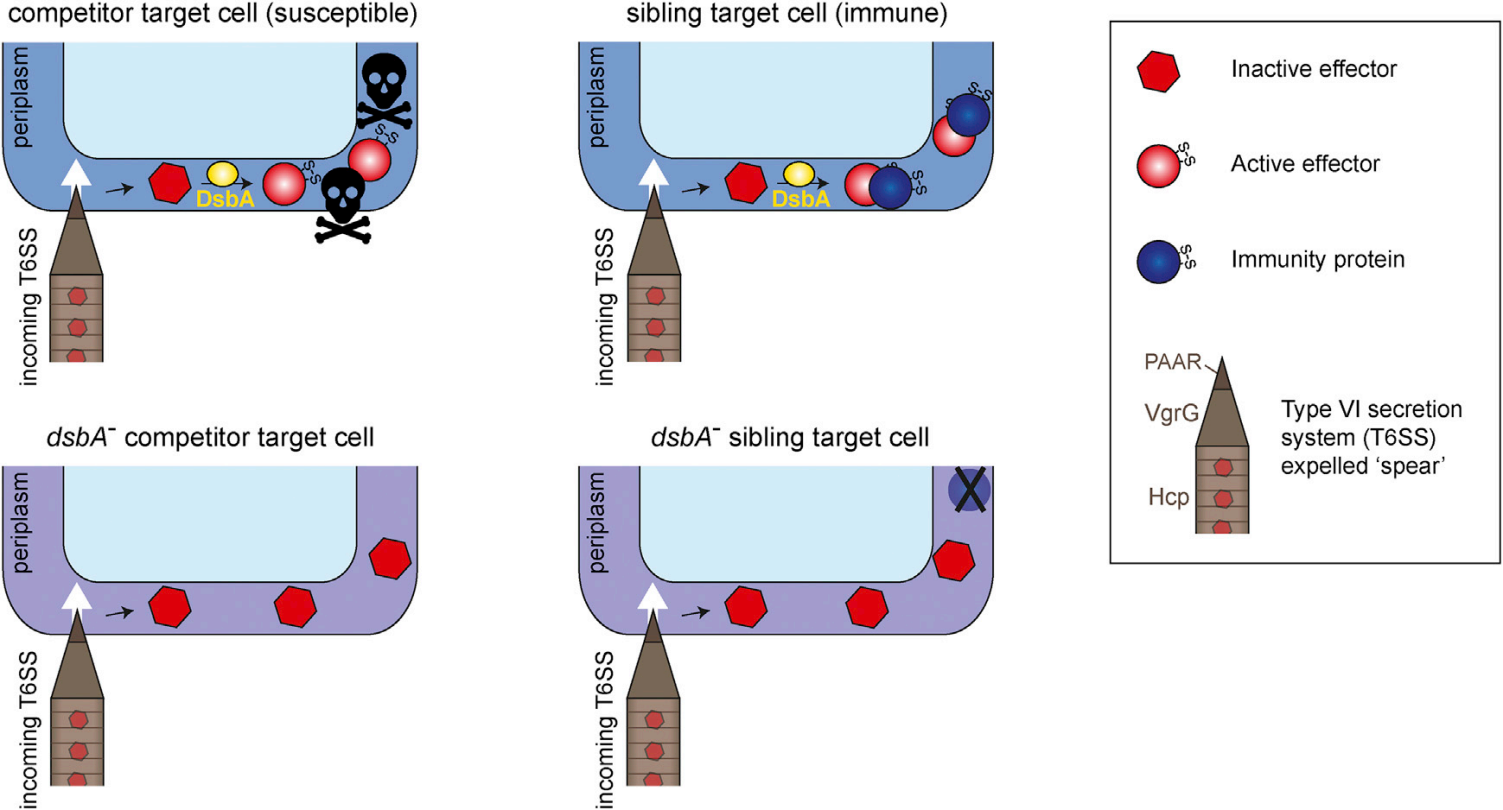
Model for DsbA-Dependent T6SS Effector Activation in Target Cells When immature effectors are delivered by the T6SS into the periplasm of target cells containing DsbA (top), the recipient cell’s DsbA machinery catalyzes the introduction of disulfide bonds into incoming effectors, allowing them to adopt their final, active conformation. If the target is a non-self-competitor (top left), the active effector causes intoxication, for example, cell wall cleavage. If the target is a sibling cell (top right), the corresponding immunity protein intercepts the effector, preventing toxicity. Alternatively, when effectors that normally require disulfide bond formation are delivered into a competitor target cell lacking DsbA (bottom left), they remain in the inactive form and do not cause toxicity. In sibling cells, cognate periplasmic immunity proteins may also require disulfide bond formation, for example, to achieve stability. However, loss of immunity protein function in a sibling cell lacking *dsbA* does not lead to intoxication, because the incoming effector is also inactive (bottom right).

The data presented here reveal that an endogenous target cell protein can play an essential role in T6SS effector activation. Incorporation of disulfide bonds by resident DsbA is required for toxicity of periplasmic effectors, exemplified by Ssp2 and Ssp4, upon their delivery into a susceptible target cell by the T6SS. A schematic model for the role of DsbA in target cell effector activation is presented in Figure 6. Considering the mechanism of effector activation, conformational change induced by disulfide bonding may affect catalytic efficiency, substrate binding, and/or stability in the periplasmic environment. In the case of Ssp2, the disulfide bond in the closely related Tae4 protein from *Ent. cloacae* is also required for toxicity and plays a role in orienting the catalytic Asp 139 residue and stabilizing the substrate binding site (Zhang et al., 2013), an arrangement apparently conserved in Ssp2 (Figure 5B). Similarly, the structure of Ssp1, a distinct member of the Tae4 family, suggests that a disulfide bond is important to form one side of its slightly different active site (Srikannathasan et al., 2013). The molecular details of DsbA contribution to Ssp4 activity are yet to be determined, however the contribution of DsbA to the activity of incoming T6SS effectors is likely to be widespread. Periplasmic immunity proteins may also contain disulfide bonds; for example, Rap1a and Rap2a, which neutralize Ssp1 and Ssp2, respectively, contain structural disulfide bonds (Srikannathasan et al., 2013). This might suggest that a DsbA mutant could be susceptible to its own effectors. However, we observed no killing of a *dsbA* mutant by the wild-type, readily explainable by the cognate effectors being simultaneously non-functional. Considering inter-species interactions, we observed that loss of DsbA in *E. coli* did confer partial resistance to the T6SS of *S. marcescens*. However, given that resistance is incomplete, presumably because of the delivery of Rhs2 and other DsbA-independent effectors, this advantage is likely to be outweighed by the fitness consequences of DsbA loss on other cellular phenotypes.

In conclusion, our findings reveal the dual importance of DsbA in T6SS-mediated antibacterial activity, both for proper functioning of the machinery in the secreting cell and also for effector activation in the target cell. The discovery that recipient cell DsbA is hijacked to activate toxicity of certain effectors represents an exciting new aspect to understanding T6SS-mediated inter-bacterial interactions. It highlights that target cell functions, in addition to the armory of the attacking cell, can determine the effectiveness of a T6SS-mediated assault, with the cumulative outcome of such interactions defining the shape of varied polymicrobial communities.

## Experimental Procedures

### Bacterial Strains and Plasmids

Bacterial strains and plasmids used in this study are described in Table S1. Mutant strains of *S. marcescens* Db10 carrying in-frame deletions or encoding His-tagged fusions proteins at the normal chromosomal location were constructed by allelic exchange using the suicide vector pKNG101 (Murdoch et al., 2011). Streptomycin (Sm)-resistant derivatives of mutant strains were obtained by transduction of the resistance allele from *S. marcescens* Db11 (English et al., 2012). The *dsbA* and *lacA* deletion mutants of *E. coli* BW25113 were obtained from the Keio collection, and the inserted kanamycin (Kn)-resistance cassettes were flipped out when required, as described (Datsenko and Wanner, 2000). Plasmids for constitutive expression of genes in *trans* were derived from pSUPROM and for arabinose-inducible expression of toxins from pBAD18-Kn, with primer sequences and details of plasmid construction provided in Table S2. Strains of *S. marcescens* were grown at 30°C, in Luria broth (LB) or in minimal media (40 mM K_2_HPO_4_, 15 mM KH_2_PO_4_, 0.1% [NH_4_]_2_SO_4_, 0.4 mM MgSO_4_, 0.2% [w/v] glucose); media were solidified with 12 g/L agar. Strains of *E. coli* were grown at 37°C in LB. When required, media were supplemented with antibiotics: ampicillin 100 μg/mL, Kn 100 μg/mL, and Sm 100 μg/mL.

### Immunodetection of Cellular and Secreted Proteins

Detection of Hcp1 and Ssp2 in cellular and secreted fractions of cultures grown for 7 hr in LB was performed as described (English et al., 2012, Murdoch et al., 2011). For detection of TssM-His, crude membrane fractions were prepared from cultures grown in 25 mL LB for 7 hr. Cells were harvested and washed in 10 mL of 50 mM Tris-HCl (pH 8), resuspended in 1 mL of 50 mM Tris-HCl (pH 8), and broken by sonication on ice. Cellular debris was removed by centrifugation (14,000 × *g*, 20 min, 4°C), then total membranes were isolated by ultracentrifugation (80,000 × *g*, 30 min, 4°C) and resuspended in SDS sample buffer (100 mM Tris-HCl [pH 6.8], 3.2% SDS, 3.2 mM EDTA, 16% glycerol, 0.2 mg/mL bromophenol blue, 2.5% β-mercaptoethanol). To detect 2269-His in whole-cell samples, cultures were grown as above, and cells were resuspended in SDS sample buffer and boiled. His_6_ immunodetection used an anti-His_5_ primary antibody (1:6,000; QIAGEN) and a horseradish peroxidase (HRP)-conjugated anti-mouse secondary antibody (1:10,000; Bio-Rad). Anti-TssJ polyclonal antibody was used as described (Rao et al., 2011).

### Co-culture Assays for T6SS-Mediated Antibacterial Activity

As described previously (Murdoch et al., 2011), the “attacker” strains of *S. marcescens* Db10 and the appropriate “target” strains were each normalized to an optical density at 600 nm (OD_600_) of 0.5, mixed at a ratio of 1:1, and co-cultured on solid LB at 30°C (or 37°C for *E. coli*) for 7.5 hr (Db10-derived targets) or 4 hr (all other targets). Target strains were Sm-resistant derivatives of the strains of interest, except for Kn^R^ mutants of *E. coli* BW25113 (Table S1). Surviving target cells were enumerated by serial dilution and viable counts on Sm (or Kn)-supplemented LB agar.

### Measurement of Bacterial Growth and Motility

Overnight cultures were diluted 1:200 in fresh media to a final volume of 180 μL and aliquoted into a 96-well plate. The plate was incubated with continuous shaking, in a Synergy 2 plate reader (BioTek), and absorbance at 600 nm (A_600_) readings were taken every 20 min. To calculate doubling times, the exponential phase of each individual replicate growth curve was used to fit an exponential trend line in Microsoft Excel and the doubling time calculated as ln(2)/c, where c is the growth rate of the population of bacteria, derived from the equation y = a × e^(c × x)^. Growth rate measurement in flasks was performed on 25 mL cultures inoculated to a starting OD_600_ of 0.02 and incubated at 200 rpm. To measure swimming motility, individual bacterial colonies were used to inoculate LB or minimal media containing 0.3% agar. Plates were incubated at 30°C for 16 hr, and the diameter of the swim halo was measured.

### Virulence Assay in *Galleria mellonella*

Virulence was assessed by determining the lethal dose of bacteria necessary to kill 80% of *Galleria mellonella* larvae (Livefoods UK) in 24 hr (LD_80_). Overnight bacterial cultures were normalized to an OD_600_ of 1 and diluted 10^−1^ to 10^−7^ in PBS. Larvae (n = 20) were injected via the hind left proleg with 10 μL of each dilution using a Hamilton syringe and incubated at 25°C for 24 hr. Larvae were then scored as live or dead, with “dead” being those that did not respond to touch. Culture dilutions were simultaneously plated on LB agar plates to enumerate the number of bacteria in the inocula.

### Fluorescence Microscopy

Five milliliter overnight cultures were diluted 1:200 in 25 mL minimal media and grown for 5 hr with shaking. Two microliters of culture were placed on a microscope slide layered with a pad of minimal media solidified by the addition of 1.5% UltraPure agarose (Invitrogen) and sealed with 1.5 thickness coverslips attached to the microscope slide with a GeneFrame (Thermo Fisher Scientific). Images were acquired using a DeltaVision Core wide-field microscope (Applied Precision) mounted on an Olympus IX71 inverted stand with an Olympus 100X 1.4 NA lens and a CoolSnap HQ2 camera (Photometrics), with differential interference contrast (DIC) and fluorescence optics. Images were acquired with the following parameters: 512 × 512 pixels, one-by-one binning, with 11 Z sections spaced by 0.25 μm. GFP and mCherry were detected using a GFP filter set (Ex 485/20 nm, Em 530/25 nm) and a mCherry filter set (Ex 542/82, Em 603/78), with exposure time of 100 ms. DIC images were acquired at 32% intensity and exposure time of 200 ms. Post-acquisition, images were deconvolved using softWoRx and stored and processed using OMERO software (Allan et al., 2012).

### Sulfhydryl Labeling in Whole Cells

Labeling of free cysteines in intact cells was performed using malPEG as described (Koch et al., 2012). Cells were grown for 7 hr in 50 mL LB, harvested by centrifugation (21,000 × *g*, 10 min), washed in 10 mL HEPES/MgCl_2_ buffer (50 mM HEPES, 5 mM MgCl_2_ [pH 6.8]), and resuspended in a volume equivalent to 10 mL for a culture harvested at OD_600_ 3.0. Labeling reactions were performed for 1 hr at room temperature, in a final volume of 100 μL HEPES/MgCl_2_ buffer containing 80 μL cell suspension, 5 mM malPEG, and 10 mM EDTA. Non-labeled control samples had malPEG omitted. Reactions were stopped by addition of DTT to 100 mM final concentration. Cells were isolated by centrifugation (21,000 × *g*, 5 min) and resuspended in 100 μL SDS sample buffer prior to SDS-PAGE.

### *In Silico* Analysis

Sequence alignments were performed using T-Coffee or MUSCLE (http://www.ebi.ac.uk/Tools/msa/). Structure models were generated using I-TASSER (Yang et al., 2015). Ssp2 model was further regularized by 1 ns molecular dynamics in explicit solvent followed by energy minimization (Gromacs 2016.3; Abraham et al., 2015). Structural alignments were performed in PyMol (version 1.8.6.0; Schrodinger).

### Statistical Methods

Quantitative data were analyzed and presented using GraphPad Prism, and pairwise comparison was performed using unpaired t tests (two tailed). All replicates were independent biological replicates.

## Supplemental Information

Document S1. Figures S1–S3 and Tables S1 and S2

Document S2. Article plus Supplemental Information
