# Supplementary material for: Dual Role for DsbA in Attacking and Targeted Bacterial Cells during Type VI Secretion System-Mediated Competition
Source: Cell Rep. 2018 Jan 29;22(3):774–85. doi: 10.1016/j.celrep.2017.12.075 (PMC5792426; doi:10.1016/j.celrep.2017.12.075)
Supplement: Document S1. Figures S1–S3 and Tables S1 and S2 [file mmc1.pdf]

Cell Reports, Volume *22*

Supplemental Information

Dual Role for DsbA in Attacking and Targeted Bacterial Cells during Type VI Secretion System-Mediated Competition

Giuseppina Mariano, Laura Monlezun, and Sarah J. Coulthurst

Figure S1

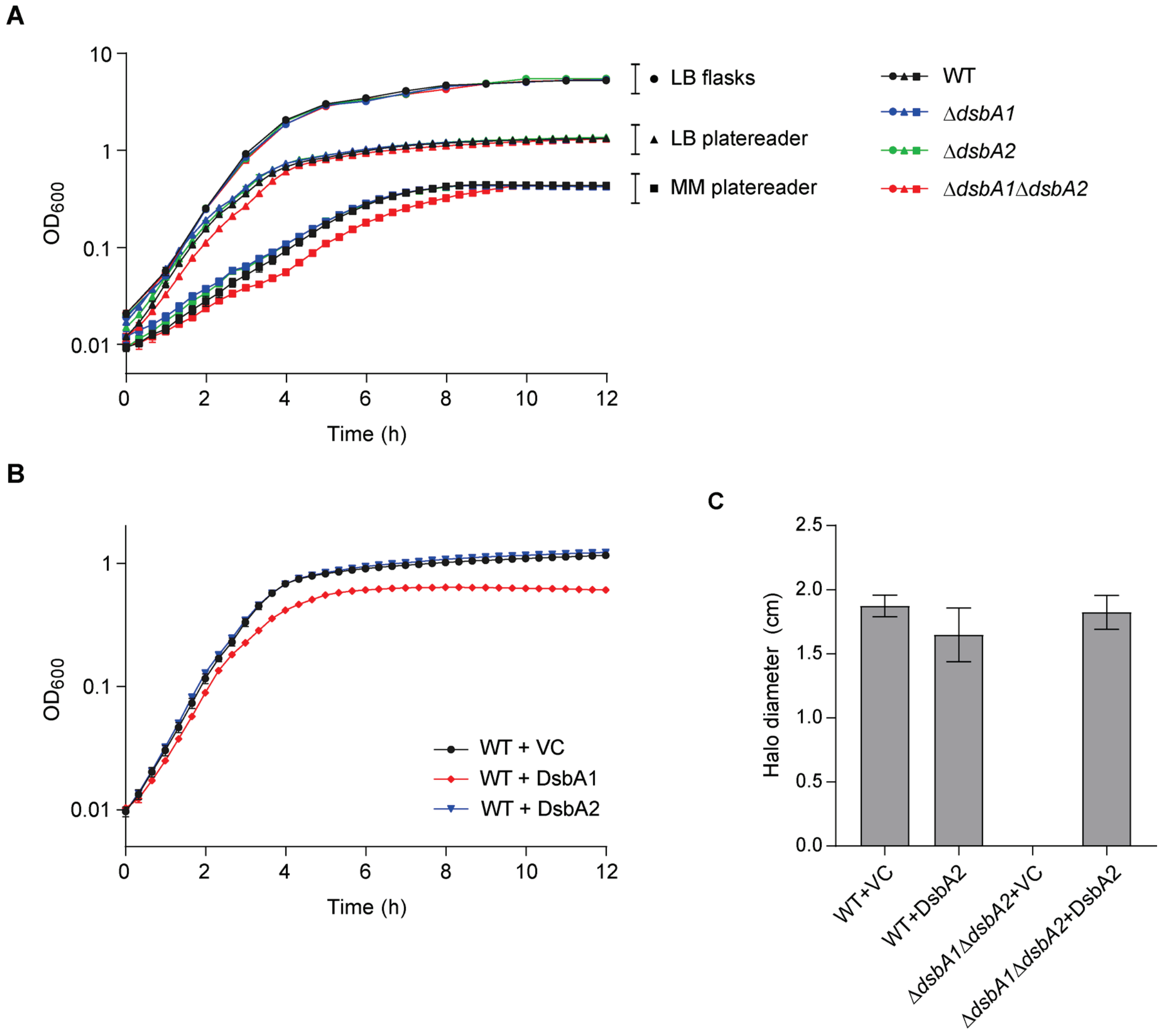

Figure S1. Growth of the single and double *dsbA* mutants and impact of DsbA1 and DsbA2 overexpression in *S. marcescens*. Related to Figure 1.
(A) Growth curves of wild type (WT) and mutant strains (Δ*dsbA1*, Δ*dsbA2* and Δ*dsbA1*Δ*dsbA2*) of *S. marcescens* Db10 with good aeration (flasks) or with limited aeration (platereader), in either rich (LB) or minimal (MM) media. Points show mean +/- SEM (n=3). (B) Growth of wild type *S. marcescens* Db10 carrying the vector control (+VC, pSUPROM) or plasmids directing the expression of DsbA1 (+DsbA1, pSC1506) or DsbA2 (+DsbA2, pSC1507) *in trans* in LB. Points show mean +/- SEM (n=4). (C) Complementation of the swimming motility phenotype of the Δ*dsbA1*Δ*dsbA2* mutant in minimal media by the expression of DsbA2 *in trans*. Bars show mean diameter of the swimming halo +/- SEM (n=4).

Figure S2

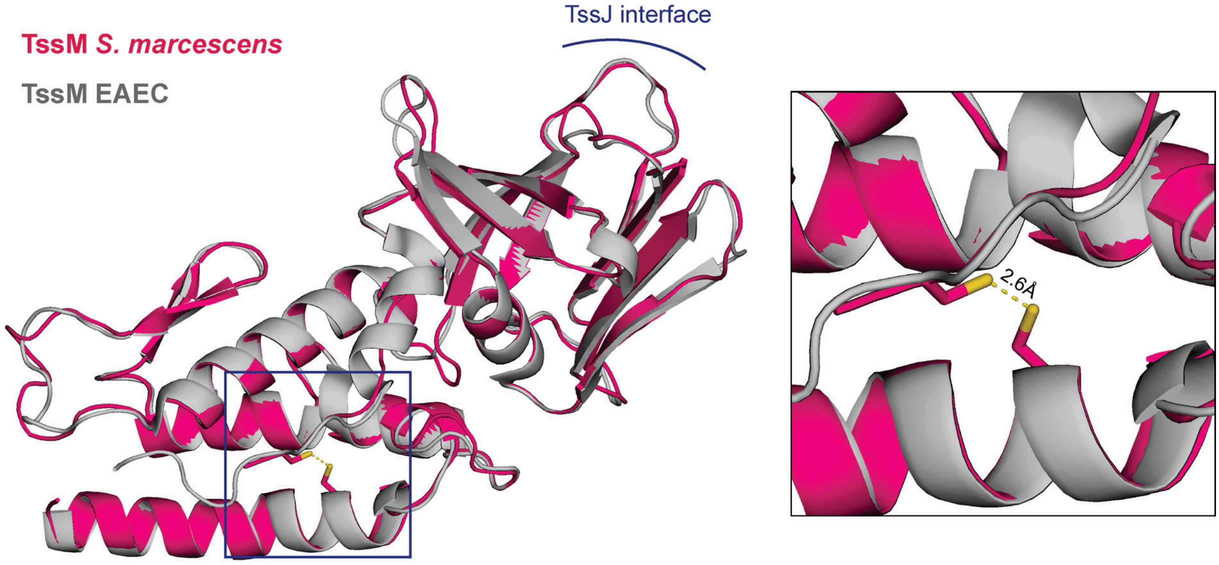

Figure S2. Structural model of the periplasmic domain of TssM from *S. marcescens*. Related to Figure 3. Cartoon representation of a structural alignment between the solved structure of the C-terminal region of the periplasmic domain of TssM from *E. coli* (aa 869-1107 out of 1129; PDB 4Y7O) and a structural model of the equivalent region of TssM from *S. marcescens* (SMDB11_2255; aa 979-1211, out of 1211). This region of *S. marcescens* TssM was identified as corresponding to PDB 4Y7O by a HHPRED search of the structural database using full length SMDB11_2255 as query (Probability 100.0, E-value 3.0E-38, 21% identity) with associated PSIPRED secondary structure prediction. The *S. marcescens* TssM model was obtained by using I-TASSER, with the *E. coli* TssM structure as a template (C score 1.79, TM score 0.97 ± 0.05) and the alignment was generated using PyMol. In the close-up view on the right, the two cysteines in the periplasmic domain of *S. marcescens* TssM (aa 998 and 1210) are represented as sticks, with yellow dashes to trace the distance between their sulfhydryl groups.

Figure S3

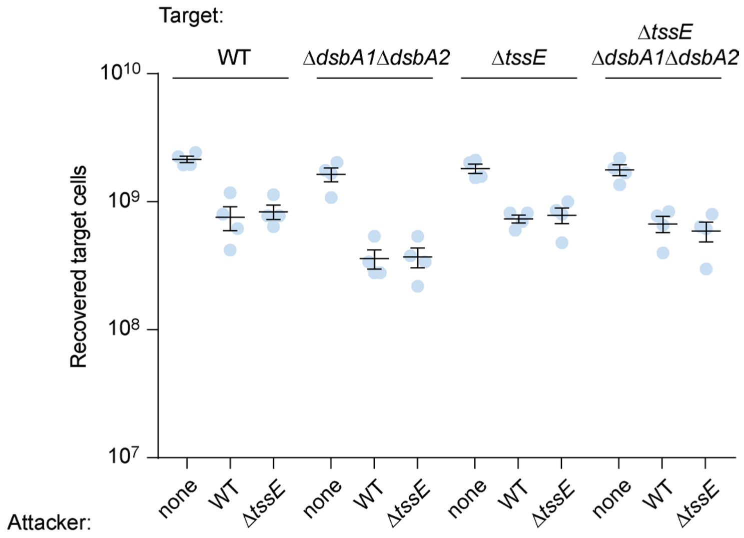

Figure S3. Loss of DsbA homologues does not cause Type VI secretion system-mediated self-toxicity. Related to Figure 4.

Recovery of wild type *S. marcescens* Db10 (WT) and the mutants Δ*dsbA1*Δ*dsbA2*, Δ*tssE* and Δ*tssE*Δ*dsbA1*Δ*dsbA2* as target strains, when co-cultured with wild type or Δ*tssE* (T6SS-inactive mutant) as attacker. 'None' indicates culture of the relevant target strain with sterile media alone and all targets were streptomycin-resistant derivatives of the appropriate strains. Individual data points are overlaid with the mean +/- SEM (n=4).

Table S1. Bacterial strains and plasmids, related to Experimental Procedures.

Name	Description	Reference
**Strains**		
Serratia marcescens		
Db10	Wild type	(Flyg et al., 1980)
SJC11	Db10 Δ*tssE* (*SMDB11_2271*)	(Murdoch et al., 2011)
GM078	Db10 Δ*tssE*, Sm-resistant derivative	This study
GM03	Db10 Δ*dsbA1* (*SMDB11_4101*)	This study
GM02	Db10 Δ*dsbA2* (*SMDB11_0239*)	This study
GM09	Db10 Δ*dsbA1*Δ*dsbA2*	This study
GM010	Db10 Δ*dsbA1*Δ*dsbA2*, Sm-resistant derivative	This study
GM030	Db10 Δ*dsbA1*Δ*dsbA2*Δ*tssE*	This study
GM031	Db10 Δ*dsbA1*Δ*dsbA2*Δ*tssE*, Sm-resistant derivative	This study
SAN199	Db10 Δ*lacZ*::P$_{T5}$-*gfpmut2*-kan^R, *tssB-mCherry* (encodes TssB-mCherry translational fusion at the native chromosomal location [*SMDB11_2258*]; cytoplasmic GFP)	(Gerc et al., 2015)
GM013	Db10 *tssB-mCherry,* Δ*lacZ*::PT5-*gfpmut2*-kan^R, Δ*dsbA1*Δ*dsbA2*	This study
JAD13	Db10 Δ*rhs2* (*SMBD11_1610*)	(Alcoforado and Coulthurst, 2015)
JAD16	Db10 Δ*rhs2*Δ*rhsI2* (*SMBD11_1610*, *SMDB11_1611*)	(Alcoforado and Coulthurst, 2015)
JAD17	Db10 Δ*rhs2*Δ*rhsI2*, Sm-resistant derivative	(Alcoforado and Coulthurst, 2015)
GM012	Db10 Δ*rhs2*Δ*rhsI2,* Δ*dsbA1*Δ*dsbA2*	This study
GM016	Db10 Δ*rhs2*Δ*rhsI2,* Δ*dsbA1*Δ*dsbA2*, Sm-resistant derivative	This study
MJF8	Db10 Δ*ssp4* (*SMBD11_3980*)	(Fritsch et al., 2013)
JAD01	Db10 Δ*ssp4*Δ*sip4* (*SMBD11_3980, SMDB11_3979*)	(Fritsch et al., 2013)
JAD06	Db10 Δ*ssp4*Δ*sip4*, Sm-resistant derivative	(Fritsch et al., 2013)
GM015	Db10 Δ*ssp4*Δ*sip4*, Δ*dsbA1*Δ*dsbA2*	This study
GM018	Db10 Δ*ssp4*Δ*sip4*, Δ*dsbA1*Δ*dsbA2*, Sm-resistant derivative	This study
SJC32	Db10 Δ*ssp2* (*SMBD11_2264*)	(English et al., 2012)
SJC43	Db10 Δ*ssp2*Δ*rap2a* (*SMBD11_2264*, *SMBD11_2265*)	(English et al., 2012)
KT63	Db10 Δ*ssp2*Δ*rap2a*, Sm-resistant derivative	(English et al., 2012)
GM014	Db10 Δ*ssp2*Δ*rap2a* Δ*dsbA1*Δ*dsbA2*	This study
GM017	Db10 Δ*ssp2*Δ*rap2a* Δ*dsbA1*Δ*dsbA2*, Sm-resistant derivative	This study
BH03	Db10 2251-His (encodes SMDB11_2251 with a C-terminal His$_6$ tag at the native chromosomal location)	This study
GM079	Db10 2251-His, Δ*dsbA1*Δ*dsbA2*	This study

GM080	Db10 TssM-His (encodes TssM [SMDB11_2255] with a C-terminal His_6 tag at the native chromosomal location)	This study
GM081	Db10 TssM-His, Δ*dsbA1*Δ*dsbA2*	This study
GM083	Db10 2269-His (encodes SMDB11_2269 with a C-terminal His_6 tag at the native chromosomal location)	This study
GM084	Db10 2269-His, Δ*dsbA1*Δ*dsbA2*	This study
Pseudomonas fluorescens		
KT02	*P. fluorescens* 55, Sm-resistant derivative	(Murdoch et al., 2011)
Escherichia coli		
MC4100	Model K-12 strain; Sm-resistant (*rpsL150*)	(Casadaban and Cohen, 1979)
CC118λ*pir*	Cloning host and donor strain for pKNG101-derived allelic exchange plasmids (λ*pir*)	(Herrero et al., 1990)
HH26 pNJ5000	HH26 Mobilizing strain for conjugal transfer	(Grinter, 1983)
BW25113	Parental strain of the Keio collection. Δ(*araD-araB*)*567*, Δ*lacZ4787 (::rrnB-4), lacIp-4000(lacI^Q), λ$^-$, rpoS369(Am), rph-1,* Δ*(rhaD-rhaB)568, hsdR514*	(Datsenko and Wanner, 2000)
-	BW25113 Δ*lacA*::Kn^R (*b0342*) from Keio collection	(Datsenko and Wanner, 2000)
-	BW25113 Δ*dsbA*::Kn^R (*b3860*) from Keio collection	(Datsenko and Wanner, 2000)
GM020	BW25113 Δ*dsbA* (*b3860*)	This study
**<u>Plasmids</u>**		
pSUPROM	Vector for constitutive expression of cloned genes under the control of the *E. coli tat* promoter (Kn^R)	(Jack et al., 2004)
pBAD18-Kn	Arabinose-inducible expression vector (Kn^R); gene of interest is cloned downstream of the P_{ara} promoter	(Guzman et al., 1995)
pKNG101	Suicide vector for allelic exchange (Sm^R, *sacBR, mobRK2, oriR6K*)	(Kaniga et al., 1991)
pSC1506	Coding sequence for DsbA1 (SMDB11_4101) in pSUPROM	This study
pSC1507	Coding sequence for DsbA2 (SMDB11_0239) in pSUPROM	This study
pSC1588	Coding sequence for Ssp2 (SMDB11_2264) in pSUPROM	This study
pSC1589	Coding sequence for Ssp2 C144A in pSUPROM	This study
pSC138	Coding sequence for $OmpA_{SP}$-Ssp2 (SMDB11_2264) fusion protein in pBAD18-Kn	(English et al., 2012)
pSC144	Coding sequences for $OmpA_{SP}$-Ssp2 + Rap2a + Rap2b (SMDB11_2264, SMDB11_2265, SMDB11_2266) in pBAD18-Kn	(English et al., 2012)
pSC1234	Coding sequence of $OmpA_{SP}$-Ssp4 (SMBD11_3980) in pBAD18-Kn	(Fritsch et al., 2013)
pSC861	Coding sequence of $OmpA_{SP}$-Ssp4 + Sip4 (SMDB11_3980, SMDB11_3979) in pBAD18-Kn	(Fritsch et al., 2013)
pSC836	Coding sequence of Ssp4 + Sip4 (SMBD11_3980, SMDB11_3979) in pBAD18-Kn	(Fritsch et al., 2013)
pSC1598	Coding sequence of Ssp4 C47A + Sip4 in pBAD18-Kn	This study

pSC1599	Coding sequence of Ssp4 C123A + Sip4 in pBAD18-Kn	This study
pSC2500	Coding sequence of Ssp4 C165A + Sip4 in pBAD18-Kn	This study
pSC1504	pKNG101-derived allelic exchange plasmid for the generation of chromosomal in-frame Δ*SMDB11_0239* (Δ*dsbA2)* deletion	This study
pSC1505	pKNG101-derived allelic exchange plasmid for the generation of chromosomal in-frame Δ*SMDB11_4101* (Δ*dsbA1)* deletion	This study
pSC1989	pKNG101-derived allelic exchange plasmid for the incorporation of *SMDB11_2251-His*$_6$ allele at the normal chromosomal location	This study
pSC1561	pKNG101-derived allelic exchange plasmid for the incorporation of *SMDB11_2269-His*$_6$ allele at the normal chromosomal location	This study
pSC1567	pKNG101-derived allelic exchange plasmid for the incorporation of *tssM-His* (*SMDB11_2255-His*$_6$) allele at normal chromosomal location	This study

Table S2. Oligonucleotide primers for plasmid construction, related to Experimental Procedures

Plasmid	Sequence of relevant primer (5'-3')	Description
pSC1504	TATATCTAGACAGGCAATGAATACCAATCAGTG	Forward primer to clone upstream region of SMDB11_0239 in pKNG101 (*Xba*I)
	TATAAAGCTTCAACATAAACACTCCTGGTTTCATC	Reverse primer to clone upstream region of SMDB11_0239 in pKNG101 (*Hin*dIII)
	TATAAAGCTTGACAAACAGCCGTAAACCG	Forward primer to clone downstream region of SMDB11_0239 in pKNG101 (*Hin*dIII)
	TATAGTCGACGAGGAAGCCCAACCGTTC	Reverse primer to clone downstream region of SMDB11_0239 in pKNG101 (*Sal*I)
pSC1505	TATATCTAGAGAGATAGACAATCTGGATCAGTTGG	Forward primer to clone upstream region of SMDB11_4101 in pKNG101 (*Xba*I)
	TATAGTCGACTTTCATAAGACTAAACTCTCCATTGTG	Reverse primer to clone upstream region of SMDB11_4101 in pKNG101 (*Sal*I)
	TATAGTCGACCAGCAGAAATAAGCCTGATTCAAC	Forward primer to clone downstream region of SMDB11_4101 in pKNG101 (S*al*I)
	TATAGGGCCCTGGTCAGACATTGTCGTGTTG	Reverse primer to clone downstream region of SMDB11_4101 in pKNG101 (*Apa*I)
pSC1561	TATATCTAGACAAACAGGAGGCCGTCGAG	Forward primer to clone upstream region of SMDB11_2269 in pKNG101 (*Xba*I)
	TATAGTCGACTCAGTGATGATGATGATGATGGTGAGT GGCGCCGTTAACCGACAG	Reverse primer to clone upstream region of SMDB11_2269 in pKNG101 (*Sal*I), incorporating His_6 tag
	TATAGTCGACTTATAAGGAAAGCCTGTGAAATCATTA GC	Forward primer to clone downstream region of SMDB11_2269 in pKNG101 (*Sal*I)
	TATAAAGCTTCGGGCAGTTGGCATACCTGTTCG	Reverse primer to clone downstream region of SMDB11_2269 in pKNG101 (*Hin*dIII)
pSC1567	TATATCTAGAGGCAGGAAGTGACGCC	Forward primer to clone upstream region of SMDB11_2255 in pKNG101 (*Xba*I)
	TATAGGATCCCTAGTGATGATGATGATGATG CGGGCACGAGAAGGC	Reverse primer to clone upstream region of SMDB11_2255 in pKNG101 (*Bam*HI), incorporating His_6 tag
	TATAGGATCCCCCCGAAAGGACATGACCATGAG	Forward primer to clone downstream region of SMDB11_2255 in pKNG101 (*Bam*HI)
	TATAACTAGTCGGCCTGATTGGTCCACCAG	Reverse primer to clone downstream region of SMDB11_2255 in pKNG101 (*Spe*I)
pSC1989	TGTATCTAGACTCGGCATGCGCATCGTGCC	Forward primer to clone upstream region of SMDB11_2251 in pKNG101 (*Xba*I)
	TATAAAGCTTCTGGAGGATCCAGGTGGCTG	Reverse primer to clone upstream region of SMDB11_2251 in pKNG101 (*Hin*dIII)
	TATAAAGCTTCATCATCATCATCATCACTAGTCAGCA CGCCTGCGGGC	Forward primer to clone downstream region of SMDB11_2251 in pKNG101 (*Hin*dIII), incorporating His_6 tag
	TATAGGGCCCTACTTCGCTTCAGCGCAGCGGTAGG	Reverse primer to clone downstream region of SMDB11_2251 in pKNG101 (*Apa*I)
pSC1506	TATAGGATCCATGAAAAAAATATGGTTGGCGC	Forward primer to clone SMDB11_4101 in pSUPROM (*Bam*HI)
	TATATCTAGATTATTTCTGCTGGCTGAGGAATTTAAC	Reverse primer to clone SMDB11_4101 in pSUPROM (*Xba*I)
pSC1507	TATAGGATCCATGTTGGCAAAAGTTAAGCGTTC	Forward primer to clone SMDB11_0239 in pSUPROM (*Bam*HI)

	TATATCTAGATTACGGCTGTTTGTCCAACAG	Reverse primer to clone SMDB11_0239 in pSUPROM (*Xba*I)
pSC1588	TATAGGATCCATGAGTCGCCCTTCATTCCAAC	Forward primer to clone SMDB11_2264 in pSUPROM (*Bam*HI)
	TAGAGCATGCGGATTTTATTTTAGTAACCATATAGATGCCTCG	Reverse primer to clone SMDB11_2264 in pSUPROM (*Sph*I)
pSC1589	TATAGGATCCATGAGTCGCCCTTCATTCCAAC	Forward primer to clone SMDB11_2264 C144A in pSUPROM (*Bam*HI)
	TATATCTAGATTATTTTAGTAACCATATAGATGCCTCGTTGGATTTTGGAAAGTACGCATGATCTGAGC	Reverse primer to clone SMDB11_2264 C144A in pSUPROM (*Xba*I)
pSC1598	Synthetic insert produced by Invitrogen GeneArt (ThermoFisher): Comprises the first 438 bp of SMDB11_3980 (nt 1-438) incorporating the C74A point mutation (codon TGC to GCG). Cloned into pSC836 using restriction sites *Sac*I-*Kpn*I, with the *Kpn*I site naturally present in the SMDB11_3980 gene sequence.	
pSC1599	Synthetic insert produced by Invitrogen GeneArt (ThermoFisher): Comprises the first 438 bp of SMDB11_3980 (nt 1-438) incorporating the C123A point mutation (codon TGT to GCG). Cloned into pSC836 using restriction sites *Sac*I-*Kpn*I, with the *Kpn*I site naturally present in the SMDB11_3980 gene sequence.	
pSC2500	Synthetic insert produced by Invitrogen GeneArt (ThermoFisher): Comprises a 184 bp internal region of SMDB11_3980 (nt 432-616) incorporating the C165A point mutation (codon TGC to GCG). Cloned into pSC836 using restriction sites *Kpn*I-*Msc*I, with the *Kpn*I and *Msc*I sites naturally present in the SMDB11_3980 gene sequence.	

Restriction sites in primer sequences are underlined.

Supplemental References

Alcoforado, D.J., and Coulthurst, S. (2015). Intraspecies Competition in *Serratia marcescens* Is Mediated by Type VI-Secreted Rhs Effectors and a Conserved Effector-Associated Accessory Protein. J Bacteriol *197*, 2350-2360.

Casadaban, M.J., and Cohen, S.N. (1979). Lactose genes fused to exogenous promoters in one step using a Mu-lac bacteriophage: in vivo probe for transcriptional control sequences. Proc Natl Acad Sci U S A *76*, 4530-4533.

Datsenko, K.A., and Wanner, B.L. (2000). One-step inactivation of chromosomal genes in *Escherichia coli* K-12 using PCR products. Proc Natl Acad Sci U S A *97*, 6640-6645.

English, G., Trunk, K., Rao, V.A., Srikannathasan, V., Hunter, W.N., and Coulthurst, S.J. (2012). New secreted toxins and immunity proteins encoded within the Type VI secretion system gene cluster of *Serratia marcescens*. Mol Microbiol *86*, 921-936.

Flyg, C., Kenne, K., and Boman, H.G. (1980). Insect pathogenic properties of *Serratia marcescens*: phage-resistant mutants with a decreased resistance to *Cecropia* immunity and a decreased virulence to *Drosophila*. Microbiology *120*, 173-181.

Fritsch, M.J., Trunk, K., Diniz, J.A., Guo, M., Trost, M., and Coulthurst, S.J. (2013). Proteomic identification of novel secreted antibacterial toxins of the *Serratia marcescens* Type VI secretion system. Mol Cell Proteomics *12*, 2735-2749.

Gerc, A.J., Diepold, A., Trunk, K., Porter, M., Rickman, C., Armitage, J.P., Stanley-Wall, N.R., and Coulthurst, S.J. (2015). Visualization of the *Serratia* Type VI secretion system reveals unprovoked attacks and dynamic assembly. Cell Rep *12*, 2131-2142.

Grinter, N.J. (1983). A broad-host-range cloning vector transposable to various replicons. Gene *21*, 133-143.

Guzman, L.-M., Belin, D., Carson, M.J., and Beckwith, J. (1995). Tight regulation, modulation, and high-level expression by vectors containing the arabinose P_{BAD} promoter. J Bacteriol *177*, 4121-4130.

Herrero, M., de Lorenzo, V., and Timmis, K.N. (1990). Transposon vectors containing non-antibiotic resistance selection markers for cloning and stable chromosomal insertion of foreign genes in gram-negative bacteria. J Bacteriol *172*, 6557-6567.

Jack, R.L., Buchanan, G., Dubini, A., Hatzixanthis, K., Palmer, T., and Sargent, F. (2004). Coordinating assembly and export of complex bacterial proteins. EMBO J *23*, 3962-3972.

Kaniga, K., Delor, I., and Cornelis, G.R. (1991). A wide-host-range suicide vector for improving reverse genetics in gram-negative bacteria: inactivation of the *blaA* gene of *Yersinia enterocolitica*. Gene *109*, 137-141.

Murdoch, S.L., Trunk, K., English, G., Fritsch, M.J., Pourkarimi, E., and Coulthurst, S.J. (2011). The opportunistic pathogen *Serratia marcescens* utilizes Type VI secretion to target bacterial competitors. J Bacteriol *193*, 6057-6069.
